# Microwave Catheter Navigation System for the Radiofrequency Liver Ablation

**DOI:** 10.3390/cancers14215296

**Published:** 2022-10-27

**Authors:** Jakub Kollar, Tomas Drizdal, Jan Vrba, David Vrba, Tomas Pokorny, Marek Novak, Ondrej Fiser

**Affiliations:** Faculty of Biomedical Engineering, Czech Technical University in Prague, 160 00 Prague, Czech Republic

**Keywords:** UWB radar, microwave imaging, medical imaging, catheter position determination, radiofrequency ablation, hepatocellular carcinoma, delay-and-sum

## Abstract

**Simple Summary:**

Hepatocellular carcinoma (HCC) is the fifth most common malignancy. Thermal ablation is one of the options for the treatment of HCC. Thermal ablation uses interstitial catheters to treat liver tumors. Catheter navigation is essential for the safety of the treatment. This work explores the possibility of tracking the catheter position during ablation treatment of HCCs using an ultra-wideband (UWB) antenna array and microwave radar imaging based on the “Delay and Sum” (DAS) algorithm. The system can track the catheter path with an accuracy of 3.88 ± 0.19 mm for simulated data and 6.13 ± 0.66 mm for experimental data.

**Abstract:**

Thermal ablation is a well-known method used in interventional radiology to treat cancer. The treatment success is closely related to the exact catheter location in the treated area. Current navigation methods are based mostly on ultrasound or computed tomography. This work explores the possibility of tracking the catheter position during ablation treatment of hepatocellular carcinomas (HCC) using an ultra-wideband (UWB) antenna array and microwave radar imaging based on the “Delay and Sum” (DAS) algorithm. The feasibility was first numerically studied on a simple homogeneous liver model. A heterogeneous anthropomorphic 3D model of the treated region consisting of the main organs within the treated area was then used. Various standard radiofrequency ablation (RFA) catheters were placed virtually in the heterogeneous model. The location and orientation of the antenna elements of the developed imaging system and the applied frequency band were studied. Subsequently, an experimental setup consisting of a 3D printed homogeneous anthropomorphic model, eight UWB dipole antennas, and catheters was created and used in a series of measurements. The average accuracy determining the catheter position from simulated and experimental data was 3.88 ± 0.19 and 6.13 ± 0.66 mm, which are close to the accuracy of clinical navigation systems.

## 1. Introduction

Cancer is one of the diseases with the highest worldwide mortality rates. However, overall cancer mortality is decreasing year by year [1]. The development of several advanced cancer treatment techniques since 1990 has contributed significantly to this downward trend [2]. One such technique is thermal ablation, in which the temperature of tumor tissue is elevated to the therapeutic values using various energy sources [3,4]. Thermal ablation uses interstitial catheters to treat tumors in multiple organs, such as the liver, lungs, kidneys, bone, and breast [3,5]. Hepatocellular carcinoma (HCC) is the fifth most common malignancy, and the standard treatment is surgical resection. Ablation treatment is an alternative to resection and is also currently and commonly used in clinical practice [6].

Nowadays, it is possible to use several techniques of thermal ablation (e.g., radiofrequency ablation (RFA), microwave ablation (MWA), ultrasound and laser ablation, etc.). RFA is based on the thermal effects of high-frequency current with an operating frequency of hundreds of kHz [4]. Compared to RFA and other types of thermal ablation, MWA is a relatively new ablation method that can treat HCC similarly to using RFA. MWA uses microwaves (MW) in the frequency range from 900 to 2450 MHz [5,7,8]. An ablation catheter is an essential part of these methods used in both RFA and MWA. The treatment of HCC is often performed with a so-called interstitial ablation catheter inserted into the treated area percutaneously, i.e., invasively. However, both methods require a navigation system to monitor the position of these catheters [9,10,11].

In interventional radiology (IR), determining the exact position of the ablation catheter within the treated area is crucial for treatment success and safety [9,12]. For this reason, an effort is made to develop increasingly accurate navigation systems with corresponding sensitivity and resolution [12,13]. The most used navigation methods for monitoring catheter position include ultrasonography (US), especially in the so-called brightness (B) mode, and CT fluoroscopy (CTF) [9,11,12,13,14,15]. The main advantages of the US include affordability, portability, real-time navigation, and, in particular, the fact that this method does not use ionizing radiation [9,13,14]. On the contrary, the main disadvantages are the dependence on the surgeon’s experience, artifacts caused by microbubbles formed during ablation, the impossibility of multiplanar imaging, blind spots behind bone structures, etc. [13,14,16]. In contrast, CTF offers better tissue imaging that the US cannot visualize and excellent spatial resolution [14,16]. The main disadvantage of guiding a catheter using CT-based imaging systems is the radiation exposure to the patient and the surgical room staff. They are exposed to significant radiation doses [17,18]. The advantages of one imaging system are, at the same time, the disadvantages of the other and vice versa. For this reason, various multimodal navigation systems are being developed and tested that combine the strengths of each applied imaging method [11,12,14,15,16]. For example, a multimodal navigation system based on a combination of US and CT provides high-resolution CT and real-time US imaging. Another example of such a system is optical navigation and electromagnetic tracking of catheter position (EM tracking) [11,12,16]. Due to advances in multimodal (RFA or MWA) catheter navigation systems, it is possible to develop new methods to contribute to the best possible results of ablation therapy in oncology.

The central part of a microwave navigation system is the antenna array containing the transmitting and receiving antennas. Transmitting antennas emit EM waves into the area of interest, while receiving antennas record the incident waves affected by attenuation, phase shifts, scattering, and reflections from tissues and objects, such as catheters, in the region of interest [19]. It is necessary to achieve a relatively high resolution to image even small objects in sufficient quality. The resolution of the ultra-wideband (UWB) microwave method is based on the used frequency bandwidth [20]. The need for a relatively wide frequency band is an essential prerequisite for using UWB technology.

Various studies are currently in progress dealing with non-invasive tissue temperature monitoring by UWB radar in hyperthermia [19] or MW tomography in ablation [21,22,23]. Progress has also been made in the field of MW detection of breast tumors [24,25,26,27], in UWB radar tomography [28], and the field of stroke detection by MW tomography [29,30]. Based on the considerable advances in microwave imaging, it can be assumed that this technology could be used to determine the exact position of the ablation catheter in the patient’s body with high precision. A catheter navigation system based on UWB radar technology would have significant advantages, such as real-time position tracking using nonionizing radiation. An array of UWB antennas makes it possible to locate the catheter position within the tissue. Another advantage is the relatively low cost required to manufacture such a system. Combining UWB radar navigation with other suitable image-based modalities could lead to a multimodal navigation system with many advantages.

This study aimed to investigate the potential of a microwave navigation system for catheter position in the HCC ablation treatment using a UWB radar system. First, we numerically studied the feasibility of this method by testing a radar system with eight UWB antennas on a simple homogeneous model and, subsequently, on a more complex anthropomorphic model. The influence of the applied frequency band (in the range of 1–10 GHz) and the influence of the position and orientation of the antennas on the detection accuracy of several ablation catheter positions were assessed in this model. The possibility of determining the trajectory of the ablation catheter during its insertion into the area of interest was also tested. Based on these numerical simulations, an experimental setup consisting of a homogeneous model and eight UWB antennas was manufactured. For three ablation catheter positions, we compared catheter reconstruction from measured and simulation data, demonstrating the potential of MW reconstruction of the catheter position.

## 2. Materials and Methods

### 2.1. Signal Processing and 2D Image Reconstruction

The detection of catheter position via UWB radar imaging is generally based on the measurements of reflections created at interfaces with two dielectrically contrasting media. UWB differential radar imaging can image dynamic changes of the dielectric parameters in the region of interest. Since ablation catheters consist of metal parts, such an object represents a highly dynamic (insertion of the catheter into the area of interest) and dielectrically contrasted structure compared to soft tissues, which in principle, can be detected via UWB radar systems.

In our case, the signal of interest is the response caused by the reflection from the catheter. Due to the constant and predetermined position of the antennas (determined by, e.g., photogrammetry techniques, laser 3D reconstruction, or by placing a gyroscope sensor at each antenna) and the static background medium, it is possible to eliminate the effect of the clutter (background) by calculation a differential signal $y_{d}\left(t\right)$ using the formula:

(1) $$y_{d}\left(t\right)={\bar{y}}_{C}\left(t\right)-{\bar{y}}_{0}\left(t\right),$$

where t is the propagation time, ${\bar{y}}_{C}\;\left(t\right)$ is the mean signal captured with the catheter in the defined position, ${\bar{y}}_{0}\;\left(t\right)$ is the mean signal captured before the catheter is inserted. 

For subsequent processing, such as “delay” determination, the UWB pulses of the transmitted and differential signals must have an easily definable position in time. For this reason, we modified differential signals using the Hilbert transformation (HT) to create the Hilbert envelope $y_{E}\left(t\right)$ of the signal using the formula:

(2) $$y_{E}\left(t\right)=\left|{\tilde{y}}_{d}\left(t\right)\right|,$$

where the ${\tilde{y}}_{d}\;\left(t\right)$ symbol denotes $\mathrm{HT}(y_{d})\;\left(t\right)$.

Furthermore, due to EM wave propagation through the lossy environment of the human body, the signal intensities captured by distant receiving antennas are significantly lower than signals received by antenna elements in the immediate vicinity of the monitored object. To eliminate this effect, which would cause problems in subsequent image reconstruction, all signals were normalized by the maximum signal intensity.

A total of eight antennas (each of which acted as transmitter and receiver) were used for imaging in both the numerical and experimental parts (total acquisition of 64 signals). A 2D space-time beamformer algorithm, “Delay and Sum” (DAS), was used for reconstruction purposes [31]. This algorithm allows summing up the contributions of the signal intensities at each point $r_{0}$ captured by individual antennas at defined positions, while the knowledge of the average speed of the signal propagation through the phantom is used [31]. The resulting intensity of the given focal point $I\left(r_{0}\right)$ is given by the formula [19,31]:

(3) $$I\left(r_{0}\right)={\left(\overset{n}{\underset{i=1}{\sum }}\overset{T_{w}/2}{\underset{\tau_{w}=-T_{w}/2}{\sum }}y_{E,i}\cdot \left(t+\tau_{i}\left(r_{0}\right)+\tau_{w}\right)\right)}^{2},$$

where n is the number of channels, $y_{N,i}$ is the recorded normalized radar signal of channel i, $\tau_{i}\left(r_{0}\right)$ is the time delay of the focal point $r_{0}$, and $\tau_{w}$ is the predefined time window.

We applied the power-law filtering to suppress the influence of the so-called “side lobes” (false targets) located in the 2D reconstruction resulting from DAS. Subsequently, the intensity of the focal point was normalized by the maximum value. Individual signal processing steps were implemented in the MATLAB (MathWorks, Natick, MA, USA) environment, including the 2D image reconstruction using the DAS algorithm.

Signal processing, especially the 2D image reconstruction using the DAS algorithm is based on the knowledge of the average propagation speed within the considered region. The average permittivity $\bar{\varepsilon_{r}}$ is crucial to determine the speed of EM waves in the given environment. For both the heterogeneous and homogeneous model, an average relative permittivity $\bar{\varepsilon_{r}}$ was determined based on the time of the flight (TOF) Δt of the emitted UWB pulse between two antennas at the distance $d_{a}$, according to the formula:

(4) $$\bar{\varepsilon_{r}}=\frac{1}{\mu_{r}}\cdot {\left(\frac{\Delta t\cdot c_{0}}{d_{a}}\right)}^{2},$$

where $c_{0}$ denotes the speed of light in a vacuum and $\mu_{r}$ relative permeability (for tissues we consider $\mu_{r}=1$).

A single value of the average permittivity was considered within the entire 2D reconstruction of the given catheter position. 

### 2.2. Numerical Feasibility Study

An initial numerical study determining the feasibility of this method was performed in the Sim4Life electromagnetic (EM) field simulator (v. 7.0, Zürich Med Tech, Zurich, Switzerland). Sim4Life uses the Finite-difference time-domain method (FDTD), which can cover a wide frequency range within a single broadband simulation, which is a significant advantage in UWB radar simulation and testing. All simulations were computed using the GPU acceleration at a workstation equipped with NVIDIA GeForce RTX 3080 Ti (NVIDIA, Santa Clara, CA, USA).

#### 2.2.1. UWB Antenna for the Numerical Study

**Figure 1:**
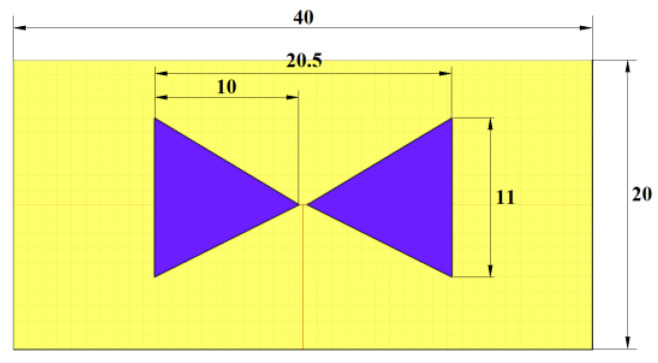
TBA model: Antenna substrate (yellow) and antenna arms (blue). All dimensions are in mm.

For the initial numerical study, we used a triangular bowtie antenna (TBA) shown in Figure 1, which was developed for UWB microwave imaging applications [32]. The antenna is built on the Rogers 4003 substrate [33], with the antenna arms modeled as a Perfect electric conductor (PEC) material. The TBA operates in the UWB frequency band of 1–10 GHz, which makes the antenna suitable for testing the influence of the UWB pulse frequency band on catheter detection accuracy. The reflection coefficient of the antenna was tested on the numerical phantom of liver tissue with the dielectric properties of $\varepsilon_{r}=38.6$ and σ=4.33 S/m set for the central frequency f=5.5 GHz [34]. The reflection coefficient was below −10 dB within the frequency band of 1–10 GHz.

#### 2.2.2. Numerical Models of Patients Torso

In our simulations, we used several phantom models with increasing complexity for several tests to determine the most optimal setup, which was manufactured and measured.

- Elliptic cylindrical model

**Figure 2:**
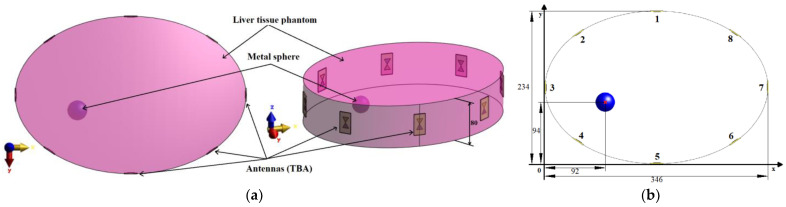
Elliptical cylindrical model: (**a**) Description of individual parts of the model; (**b**) Dimensions of the model and position of the imaged object and antenna numbers. All dimensions are expressed in mm.

For the antenna placement test, we created a simplified homogeneous elliptic cylindrical model (elliptical base dimensions were 346 × 234 mm^2^) representing the patient torso with symmetrically placed antennas in one imaging plane, see Figure 2. In the liver tissue phantom, a metal sphere with a diameter of 3 cm representing the imaging target was inserted. The dielectric parameters of the liver tissue were adjusted to a value of $\varepsilon_{r}=41.4\;\mathrm{and}\;\sigma =2.47\;S/m$, which corresponds to the liver dielectric properties at 3.5 GHz. A metal sphere represents a suitable object for this test due to its dimensions ensuring uniform reflection of EM waves and a well-defined position (see Figure 2b). The diameter of 3 cm was chosen with respect to the lowest operating frequency of 1 GHz in the liver tissue, and this diameter of the metal sphere is close to half of the wavelength at 1 GHz. For all simulations, we used absorbing boundary conditions (ABC), specifically uniaxial perfectly matched layers (UPML), ensuring EM wave absorption at the outer boundaries of the computational domain.

This elliptic model represents an ideal situation for differential imaging of the given object. It was used to optimize antenna positions to improve the reconstruction of the metallic object.

- Heterogeneous Model

**Figure 3:**
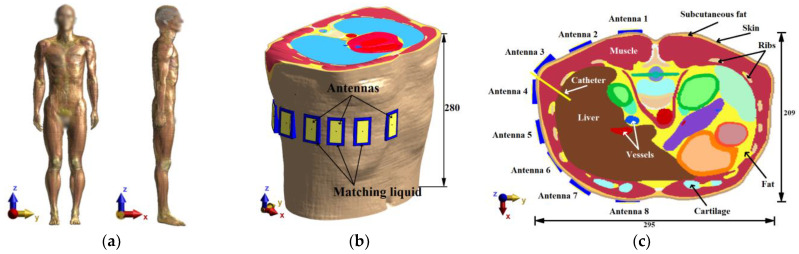
Heterogeneous model: (**a**) Duke patient’s model; (**b**) Model with placed antennas; (**c**) Imaging plane with antenna layout and description of essential structures. All dimensions are expressed in mm.

The heterogeneous model creates a more realistic scenario for testing the various radar system parameters and enables further improvements of the entire setup. As a heterogeneous model, the pre-segmented patient model “Duke” (IT IS Foundation, Zürich, Switzerland) was used [35]. The numerical model and antenna placement are shown in Figure 3a.

The results from numerical studies on the cylindrical model led to modified antenna positions, as seen in Figure 3b,c. The antennas were submerged in the compartments with the matching liquid ensuring an ideal antenna contact with the patient’s skin (without any air gaps). The test model of the ablation catheter was modeled as a long thin metallic cylinder with a diameter of 2.1 mm, which was selected with respect to the actual dimensions of commonly used RFA catheters [36,37,38].

The values of the dielectric parameters for the central frequency $f_{c}$ of the considered UWB pulses were assigned from the *IT’IS Database* [34] to the model domains corresponding to individual tissues. For completeness, these values are listed in Table 1.

- Grid settings

A rectangular grid was used to discretize the model. The model was divided into several structures with similar properties, which subsequently defined the maximum dimensions of the grid step based on the maximum frequency. The grid was refined by a factor of 1.7, the maximum achievable refinement factor allowing GPU acceleration. This grid refinement caused an average change in the monitored parameters (S_mn_) at the level of 3.32%. Due to the negligible difference in the monitored values, the refinement can be considered sufficient. The grid parameters are listed in Table 2.

#### 2.2.3. Numerical Studies

As part of the numerical study, a UWB antenna array was designed, optimized, and tested on simple elliptic cylindrical homogeneous and further on a more complex heterogeneous model. In the individual tests, the antenna positions were optimized based on the intensity of the received reflections. The antenna’s polarization and the UWB frequency band were selected with respect to the 2D reconstruction quality of the catheter positions and the detection accuracy. Following numerical tests were performed as part of the system development procedure:

- Antenna positions (Cylindrical model)

Because the treatment area is predetermined, a following simple study was chosen to determine appropriate antenna positions to monitor the position of the entire catheter. For this numerical study, the simple homogeneous elliptic cylindrical model was used. The goal of this study was to optimize the general antenna placement around the phantom, as it is shown in Figure 2. The Gaussian UWB pulses in the frequency band 1–6 GHz were sequentially transmitted from each antenna, and the amplitudes of received signals on all receiving antennas were compared and evaluated. Based on these results, the antennas were placed equidistantly around the right part of the heterogeneous model. The optimized system was subsequently used for all the subsequent numerical studies.

- Influence of Antenna Polarization (Heterogeneous model)

**Figure 4:**
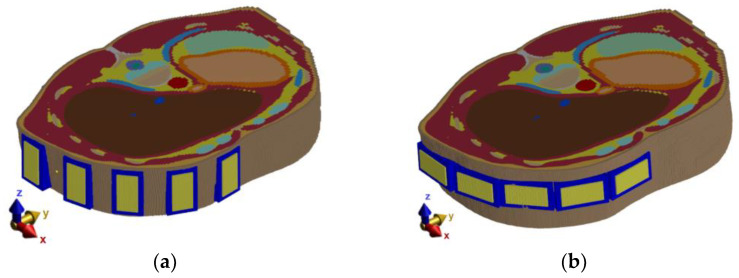
Simulation model: (**a**) Vertical placement of antennas; (**b**) Horizontal placement of antennas.

The antenna positions were changed based on the results from the previous study, as can be seen in Figure 3c. With this modified simulation setup, the influence of the antenna polarization on the reconstruction of the entire catheter position in the frequency band 1–6 GHz was studied. The relation between antenna polarization and catheter orientation within the body significantly influences the received signal intensity. Therefore, simulations were performed for vertical and horizontal antenna polarizations, as seen in Figure 4.

**Figure 5:**
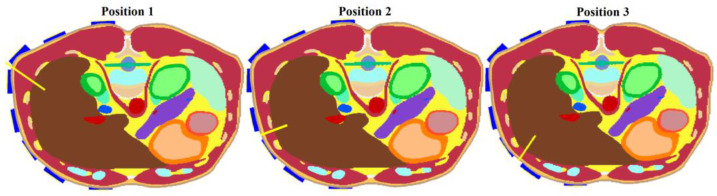
Tested positions of the RFA catheter within the heterogeneous numerical model. The solid yellow line represents the RF catheter.

After considering the results of the antenna polarization test (see Section 3.1.2.), the vertical placement of the antennas was chosen as more suitable. For further tests, models of three different RFA catheter positions were created, see Figure 5. These models were used to test the final optimized system.

- UWB Pulse Frequency Bandwidth Study (Heterogeneous model)

This study aimed to investigate the effect of the frequency bandwidth on the resulting 2D reconstruction. Three Gaussian-type UWB pulses (1–4 GHz, 1–6 GHz, and 1–10 GHz) using the heterogeneous model with the catheter in position 1 according to Figure 5 were tested, and the 2D reconstructions were carried out.

- RFA Catheter Tip Position Detection during Insertion (Heterogeneous model)

**Figure 6:**
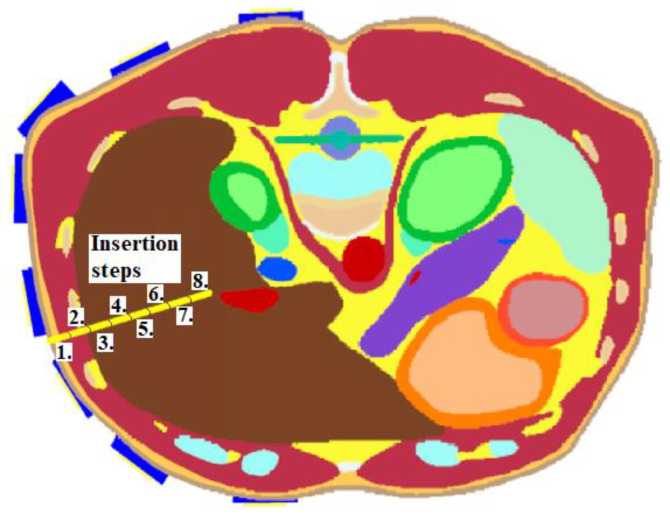
Insertion steps 1–8 of the ablation catheter.

This numerical study aimed to test the UWB radar system in the ablation catheter tip position detection during its insertion into the liver tissue. This situation represents an actual clinical situation of an ablation procedure. During this study, the entire trajectory of the ablation catheter tip was continuously monitored. To model this dynamic situation, the study was divided into eight simulations with the insertion step of 1 cm. Insertion steps 1–8 are visualized in Figure 6. This method enables the reconstruction of the catheter tip position, which was inserted in the given step. The implemented 2D reconstruction algorithm subsequently processed obtained differential signals to create a total of eight image reconstructions. These reconstructions determined the maximum intensity for each insertion step representing discrete tip points of the catheter trajectory.

### 2.3. In Vitro Experimental Measurement Setup

#### 2.3.1. Antenna for Measurement Session

**Figure 7:**
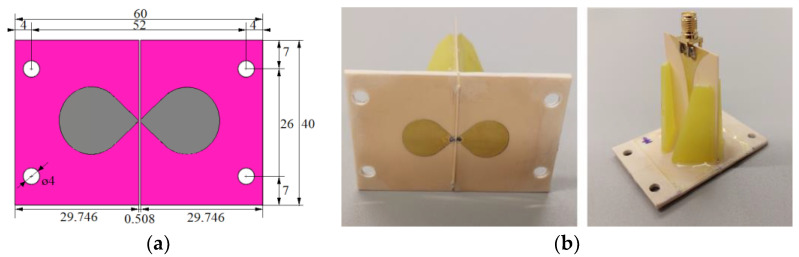
TBARC antenna: (**a**) Dimensions of the numerical model of the modified antenna; (**b**) Assembled modified TBARC antenna. All dimensions are expressed in mm.

For the in vitro measurement sessions, the new UWB triangular bowtie antenna with rounded corners (TBARC) with UWB balun operating in the frequency band 1–6 GHz was used. The choice of the improved rounded UWB bowtie antenna was made due to its good parameters for the UWB microwave imaging methods. The antenna has a symmetrical radiation pattern, low signal distortion, and high radiation efficiency toward the tissue [39]. The antenna dimensions of the front panel were modified to fit in the geometry of the patient’s torso model, see Figure 7a, however, this modification does not influence the antenna parameters. The antennas were fabricated and assembled as presented in [39]; see Figure 7b.

#### 2.3.2. Measurement-Experimental Model

**Figure 8:**
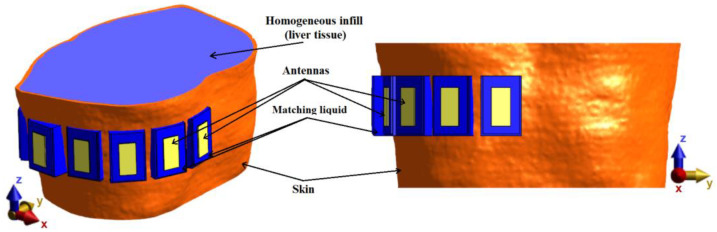
The homogeneous numerical model with a description of individual parts.

For verification of the UWB radar system, a homogeneous model based on the system proposed in previous numerical studies consisting of an anthropomorphic envelope (skin model, taken from Duke model) filled with a homogeneous liquid with dielectric parameters of liver tissue was created, and subsequently constructed. The numerical model with TBA antennas can be seen in Figure 8.

**Figure 9:**
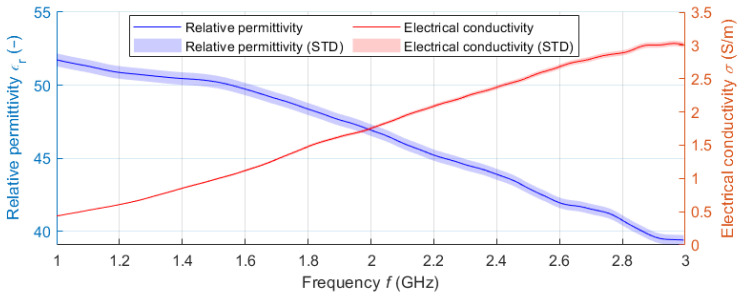
Measured frequency dependence of the dielectric parameters of the liquid liver phantom. The values are supplemented with a confidence interval corresponding to the expanded measurement uncertainty.

The filling liquid was a mixture of deionized water (59.8 wt%) and isopropanol (IPA-40.2 wt%). The dielectric parameters of the liquid phantom were measured in the frequency band 1–3 GHz using the SPEAG DAK-12 open-ended coaxial probe (Schmidt & Partner Engineering AG, Zurich, Switzerland) connected to a Fieldfox N9913A (Keysight, Santa Rosa, CA, USA) Vector Network Analyzer (VNA). Since the central frequency with which the designed UWB radar system operates is 3.5 GHz (for the 1–6 GHz band), a frequency of 2.5 GHz was chosen to adjust the dielectric parameters of the liquid phantom. The matching liquid was prepared the same way as the liver tissue liquid phantom. The individual curves (see Figure 9) represent the average values obtained within ten measurements. The shading error bars represent the ± expanded measurement uncertainty with the coverage factor *k* = 2.

**Figure 10:**
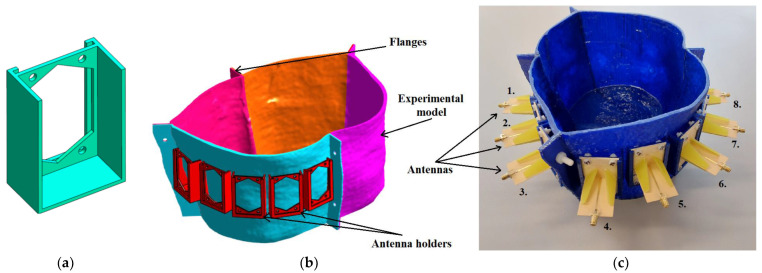
Experimental model: (**a**) Antenna holder; (**b**) Description of individual parts of the experimental model; (**c**) Assembled experimental model with placed antennas.

The experimental model used for measurement was created based on the geometry of the homogeneous numerical model and was used for the system verification and verification of the potential to track the RFA catheter using the UWB radar technique. First, antenna holders were created to ensure their exact position within the model and to accommodate matching liquid, see Figure 10a. Three-dimensional printing technology was used using the Original Prusa i3 MK3S+ (Prusa Research, Czech Republic) printer and PET-G material to make the torso of an actual patient. The model was divided into four parts (see Figure 10b), and after printing, the parts were connected using polyamide (PA) screws and epoxy glue. Antennas were fixed with PA screws and sealed with epoxy glue, see Figure 10c. To increase waterproofness, the entire model was coated with epoxy resin from the inside.

**Figure 11:**
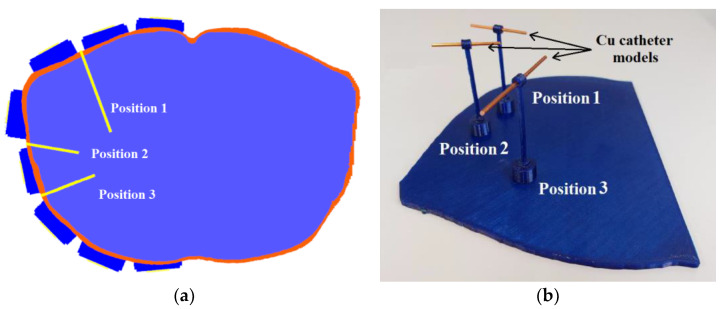
Catheter positions: (**a**) Numerical model of the treatment area with three selected catheter positions; (**b**) 3D printed structure for holding catheters’ mockups (sections of copper wire) in the corresponding positions during experiments.

Further, we designed a holder to insert three catheters on the predefined positions, see Figure 11a. A designed positional model ensured the exact catheter positions within the experimental model. This model was designed for accurate and repeatable placement of copper wires with a 2 mm diameter representing the catheters during the measurements. These catheters were placed one by one into the positional model created using 3D printing, see Figure 11b. The length of the individual copper wires was chosen considering their length in the homogeneous numerical model (for position 1–70 mm, position 2–42 mm, and position 3–45 mm).

#### 2.3.3. Measurement Setup

All eight antennas were placed in holders and connected to the VNA Rohde & Schwarz ZNB 8 (Rohde & Schwarz, Munich, Germany) through the Rohde & Schwarz ZN-Z84 switching matrix, expanding the number of usable ports. The VNA was calibrated just before the measurement using the Rohde & Schwarz ZN Z152 calibration unit in the frequency bandwidth of 1–6 GHz and with 201 frequency points. The VNA output power was set to 13 dBm and the intermediate frequency bandwidth to 100 Hz.

**Figure 12:**
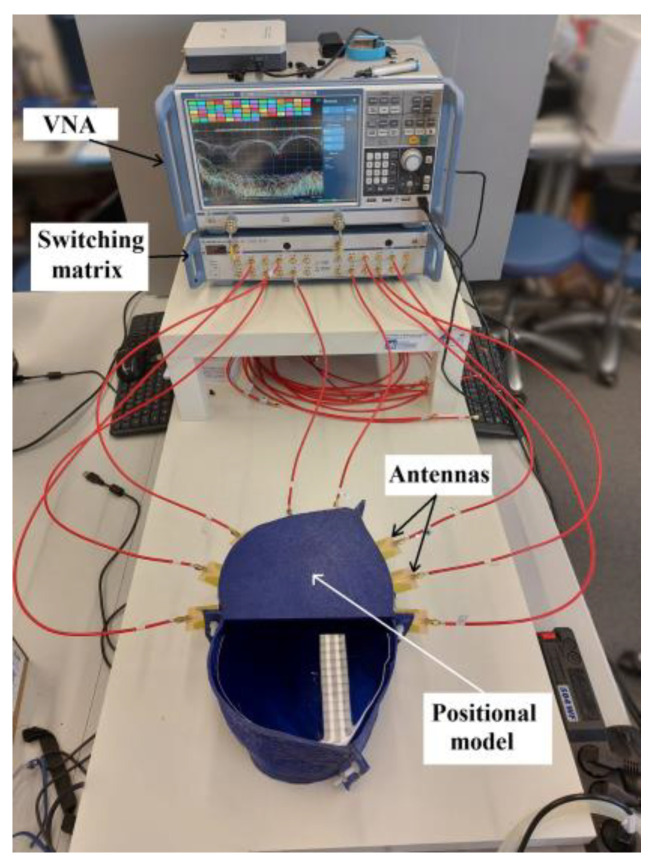
The experimental setup was used for measurements with the description of the individual parts.

The interior space of the experimental model and antenna holders was filled with a liquid phantom of the liver tissue, see Figure 12. Measurements were performed for three catheter positions with two measurements for each, one with the catheter placed in the defined position and the other without the catheter, to obtain a differential signal. The measured S-matrix was converted to the time domain using IFFT and processed using the DAS reconstruction algorithm. The subsequent numerical simulations were computed and compared with the measurements for the results verification.

### 2.4. Determining the Accuracy of Ablation Catheter Detection System

In the simulations with the cylindrical model, the imaged object was a metal sphere with a diameter of 3 cm. The position of the sphere in the model is defined by the coordinates [*x*, *y*] in the Cartesian coordinate system, see Figure 2b.

**Figure 13:**
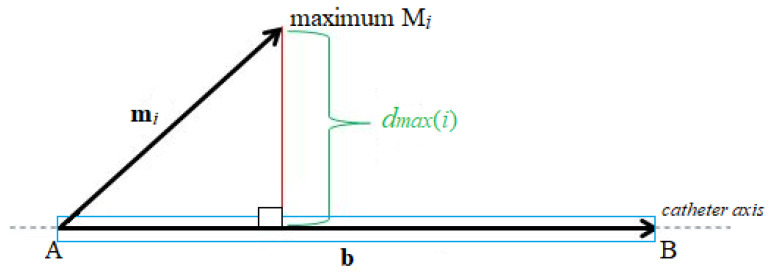
Principal scheme of the point-to-line method.

To evaluate the detection accuracy of an object such as a catheter, the point-to-line method was proposed using the determination of the perpendicular distance $d_{max}$ of individual maxima from the axis of the catheter, see Figure 13.

Determining the point-to-line distance is based on the knowledge of the position of the maximum M*_i_*, the position of the initial point A, and the final point B of the catheter axis as:

(5) $$d_{max}=\frac{1}{N_{max}}\cdot \overset{N_{max}}{\underset{i=1}{\sum }}\frac{\left\|m_{i}\times b\right\|}{\left\|b\right\|}$$

Within the method, the selected total number of maxima was set to $N_{max}=50$.

## 3. Results

### 3.1. Numerical Study

#### 3.1.1. Optimization of Antenna Positions

**Figure 14:**
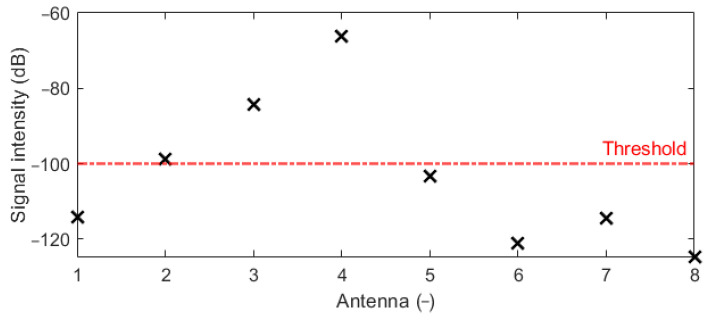
Hilbert envelope of differential signals calculated using $S_{nn}$ signals with the expected echo from the target.

**Figure 15:**
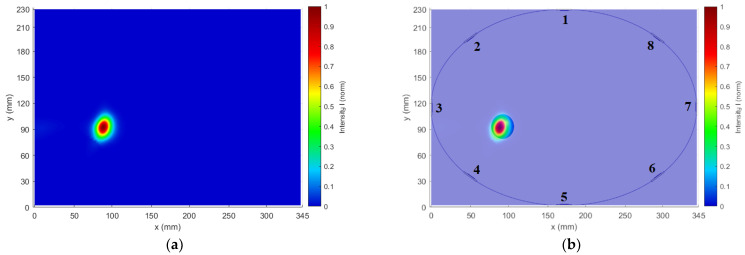
Results of 2D reconstruction: (**a**) Output of the image reconstruction algorithm; (**b**) Overlay of 2D reconstruction with the real position of the imaged object in the model. The color bar represents the normalized intensity of the focal point obtained from the DAS reconstruction algorithm according to Equation (3).

The cylindrical model with the metal sphere centered in the position [92; 94] mm was used for the antenna placement test. The received differential signal varies in amplitudes that depend on the antenna distance from the point of the reflection. Figure 14 presents amplitudes of received differential signals calculated using $S_{nn}$ signals. In Figure 14, the differential signals captured by antennas 1 and 5–8 (antenna numbering is according to Figure 2b) are below a threshold, where the signal from Antenna 8 is on the level of numerical noise. The threshold of the signal value was determined to be −100 dB between the received and emitted signal amplitude. The lower signal amplitudes reduce the reconstruction accuracy. These antennas are located too far from the object and thus provide no useful information for imaging purposes. Based on this observation, we adjusted the position of the antennas to provide a signal with higher amplitudes. The remaining signals, i.e., signals captured by antennas 2–4, were used for successful 2D reconstruction of object position, see Figure 15.

The maximum intensity in the reconstruction (Figure 15) is at the coordinates [90.56; 92.89] mm. The detection accuracy was determined as the distance of the metal sphere center from the intensity maximum, which was 1.81 mm.

#### 3.1.2. Influence of Antenna Polarization

**Figure 16:**
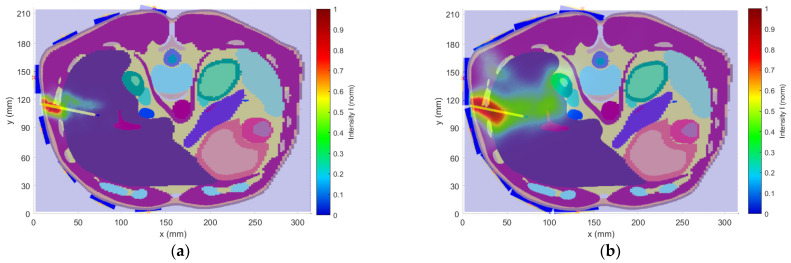
The antenna polarization study results: (**a**) Reconstruction for vertically placed antennas; (**b**) Reconstruction for horizontally placed antennas.

In the second numerical study, the influence of the antenna polarization (horizontal and vertical) on the catheter position reconstruction was tested. Figure 16 presents the reconstruction of the catheter position within a heterogeneous phantom for both polarizations. The result for vertical antenna polarization (Figure 16a) shows more accurate catheter detection with low deviation around the catheter tip. For the horizontal polarization (Figure 16b), the blur effect along the whole catheter reduces the detection accuracy.

#### 3.1.3. UWB Pulse Frequency Band Study

**Figure 17:**
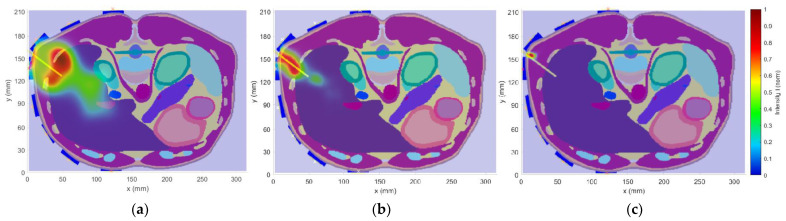
Effect of frequency bandwidth on imaging accuracy tested on the heterogeneous model with the catheter in position 1: (**a**) Frequency band 1–4 GHz; (**b**) Frequency band 1–6 GHz; (**c**) Frequency band 1–10 GHz.

The effect of the bandwidth of the UWB pulse on the detection accuracy was tested on the heterogeneous model in which the catheter on position 1 was placed. The reconstructions for each frequency band are presented in Figure 17a–c). The focal area around the catheter is shrinking with the increasing frequency bandwidth. For the frequency band 1–4 GHz in Figure 17a and 1–10 GHz in Figure 17c, the reconstructions are not usable because of lower accuracy, see Table 3. The ideal bandwidth appears to be 1–6 GHz, where the detection accuracy was 2.16 mm.

#### 3.1.4. Numerical Catheter Position Study

**Figure 18:**
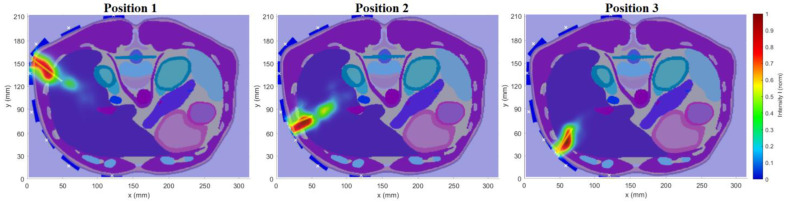
Results of 2D reconstruction for catheter position 1, 2, and 3 in the heterogeneous model.

The modified system was tested on the heterogeneous model for three different catheter positions in the following study, according to Figure 5. The results of the 2D reconstruction are presented as an image overlay with the actual position of the catheter in the given model, see Figure 18. Table 4 are presents the detection accuracy values for each catheter position.

#### 3.1.5. Detection of the RFA Catheter Tip Position during Insertion

**Figure 19:**
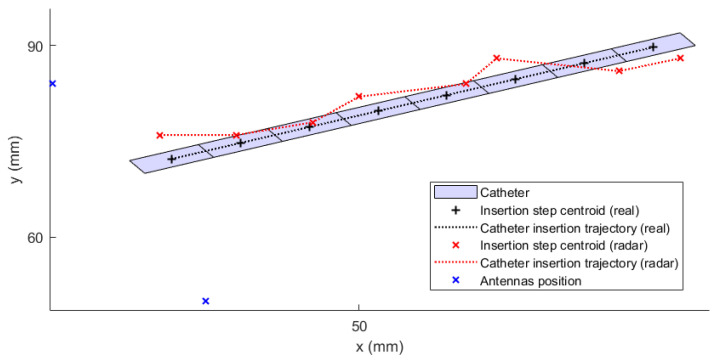
Estimated catheter tip position detection during insertion (red dotted line).

In this numerical study, we tested the ability of the optimized UWB radar system to detect the ablation catheter tip position during its insertion. The catheter tip trajectory determined by the radar and compared with the real trajectory is presented in Figure 19. In Figure 19, the red crosses represent the position determined by the radar method of the catheter tip with the estimated catheter tip trajectory marked by the red dotted line. The black crosses visualize the real position of the catheter tip in each step with the catheter trajectory marked by the dotted black line. The detection accuracy $d_{centr}$ was determined by the distance of the corresponding intensity maximum from the centroid of a given catheter step; see Table 5. The mean detection accuracy of the tip was 3.33 mm. For steps 2 and 3, the accuracy is significantly better. For the remaining steps, the accuracy remains constant.

#### 3.1.6. Determination of Average Permittivity in Heterogeneous Models

As a part of the UWB radar system testing on the heterogeneous model, the average permittivity was calculated from the time of flight (TOF) of the emitted UWB pulse. The average permittivity $\bar{\varepsilon_{r}}$ was determined based on the method described in Section 2.1. The determined $\bar{\varepsilon_{r}}$ in the considered pulse frequency bands are presented in Table 6 (third row). The specified $\bar{\varepsilon_{r}}$ determined by the TOF method is compared with the permittivity values at *f_c_* of two main tissues (liver and muscle tissue) in Table 6.

From the $\bar{\varepsilon_{r}}$ comparison presented in Table 6, the determined $\bar{\varepsilon_{r}}$ from the TOF method are in the middle of the $\varepsilon_{r}$ interval of the liver and muscle tissues for all frequency bands. The method was also verified on the homogeneous model (see Section 2.3.2) with a specific relative permittivity of liver tissue $\varepsilon_{r}=41.4$. The relative permittivity value determined by the TOF method within the homogeneous model was $\varepsilon_{r}=43.7$, which represents an acceptable deviation of 5.56% from the real value.

### 3.2. Experimental Measurement

**Figure 20:**
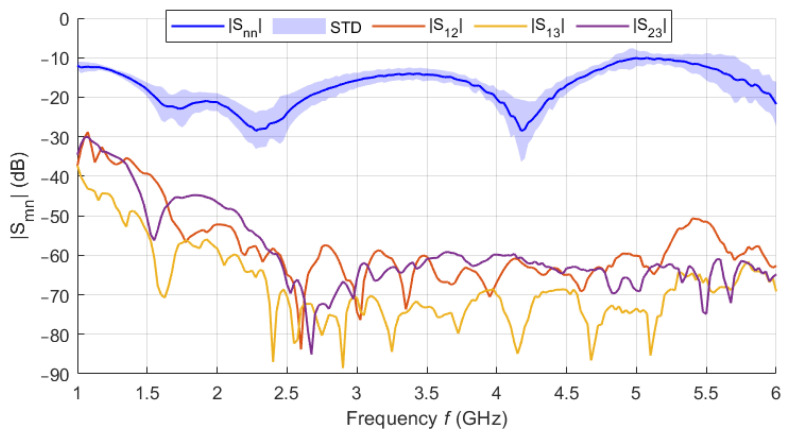
Representative examples for individual $\left|S_{\mathrm{mn}}\right|$ parameters for the catheter at position 1.

The numerically modified UWB radar system was tested on an experimental model (see Figure 10) based on the geometry of the homogeneous model with identical antenna positions (see Figure 11a). Figure 20 shows representative examples of individually measured $\left|S_{\mathrm{mn}}\right|$ parameters for the catheter at position 1. $\left|S_{\mathrm{nn}}\right|$ parameters are presented in the form of the average value of $\left|S_{\mathrm{nn}}\right|$ parameters captured by antennas 1–8, where the shading error bar represents the STD. The S-parameter matrix was processed in the way described in Section 2.3.3.

**Figure 21:**
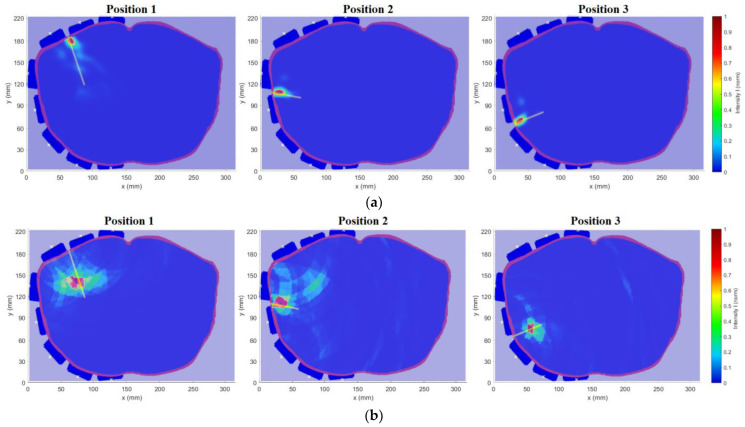
Results of 2D reconstruction for catheter position 1, 2, and 3: (**a**) Homogeneous numerical model; (**b**) Experimental model measurement.

Signals obtained from the simulations and signals in the time domain obtained from the measurements were processed by the methods presented in Section 2.1. The obtained image overlay of the 2D reconstruction with the actual position for a total of three catheter positions is shown in Figure 21.

The resulting determined detection accuracy values of the individual RFA catheter positions using the optimized UWB radar system within the considered models are presented in Table 7.

## 4. Discussion

In phantom experiments, we successfully designed and verified the potential of the catheter UWB radar navigation system for liver RFA ablation. The system consists of eight triangular bowtie antennas with rounded corners, which we obtained during experimental measurements of the |S_11_| bellow −10 dB throughout the frequency range of 1–6 GHz. These antennas were mounted on the 3D printed frame for which we used surface delineation of the Duke model from the Virtual Family models. For this system and homogeneous phantom, we obtained the average catheter detection accuracy $\;{\bar{d}}_{max}=3.88\;\pm \;0.19$ mm from the numerical simulations and ${\bar{d}}_{max}=6.13\;\pm$ 0.66 mm from experimental measurements. Predicted average detection accuracy was improved to ${\bar{d}}_{max}=2.72\;\pm$ 0.48 mm, when testing the developed system on a detailed Duke model. The ability of the system to detect the catheter insertion trajectory was demonstrated on this heterogeneous model, with an average deviation of ${\bar{d}}_{centr}=3.33\pm 1.43$ mm.

Several numerical studies were performed to obtain the best possible antenna placement, orientation, and frequency band of the UWB antennas. The first simulation study revealed that the intensities received by antennas placed at the left part of the body are below the −100 dB threshold. Because of this, the antennas were equidistantly distributed only in the right part of the patient model, i.e., around the immediate vicinity of the detected target (within the liver area). The second numerical study showed that despite the lower received signal intensities for vertically polarized antennas compared to horizontally polarized ones, the quality of the resulting image reconstruction was not significantly affected. We considered the RFA catheter position in parallel to the imaging plane, which represents the worst possible scenario for the vertical antenna polarization (see Figure 16a,b). Further, horizontally polarized antennas occupy more space and thus limit the space for catheter insertion, which is also limited by specific criteria for catheter insertion [40]. In the last numerical study, we demonstrated that with 1–6 GHz frequency bandwidth of the UWB signal achieved a relatively good image reconstruction quality and, at the same time, the best detection accuracy, i.e., $d_{max}=2.16$ mm can be achieved. Using the 1–4 GHz frequency band, on the one hand, allowed, due to the lower tissue conductivity at the central frequency of 2.5 GHz, the capture of signals with greater intensity, but at the expense of resolution (see Figure 17a). On the other hand, using the 1–10 GHz band was characterized by a relatively high resolution. Due to the high conductivity of tissues at the central frequency of this band and the associated attenuation of EM waves, information about the nature of the detection target was carried only by the signals captured by the antennas closest to the catheter. This fact resulted in a deterioration of the image reconstruction quality (see Figure 17c).

During the system tests on the heterogeneous model, it was challenging to determine the average permittivity, without which it is impossible to successfully reconstruct the catheter position. As a suitable and relatively robust method, we chose the method based on the knowledge of the TOF and the constant distances between the antennas within the system. Using this method, validated on the homogeneous model, we were able to determine the average permittivity for all frequency bands. The deviation of the real permittivity from the value determined by the TOF method was 5.56%. This deviation can be considered acceptable; therefore, the TOF method is suitable for determining the average permittivity, especially for heterogeneous models.

The detection accuracy of the proposed navigation system is comparable to the detection accuracy of commercial navigation systems. The accuracy of EM tracking was 3.2 ± 2.1 mm, depending on the methods of individual experiments, and the accuracy of CBCT was 3.83 ± 1.92 mm [11]. However, it should be noted that these values were obtained based on experiments with real navigation systems and using more accurate patient phantoms, possibly on patients. We identified the following possible issues which can occur in the case of clinical usage of our system. The patient’s movements related to breathing/heart movements can influence the system’s accuracy. We assume that these effects will be neglectable. During the RF ablation, the tissue is locally heated, and this causes changes in dielectric properties. These changes within the considered temperature range are in order of percentages [19,27]. We assume that the amplitudes of UWB signal reflections from the heated regions will be several times lower than the reflections from the metallic catheter, and the detection accuracy will not be influenced. The next issue could be mutual interference between the microwave and RFA systems. The typical RFA system works at a frequency of around 500 kHz. Our system works in the frequency band 1–6 GHz. There is no frequency overlap. We are not expecting the EMC issue between the RFA system and our UWB system. As presented, the UWB radar system achieves accuracy close to commercially used systems such as EM tracking. However, after improvements planned in the future, a single complex system could be developed that would be able to continuously track the catheter position during its insertion into the treated area and subsequently monitor the progress of the ablation, i.e., the temperature distribution in the area, as presented by several authors [21,22,23].

## 5. Conclusions

We have proposed, optimized, developed, and tested the microwave catheter navigation system for radiofrequency liver ablation based on the UWB radar. The system is capable of tracking the path and the position during the catheter insertion into the patient. The reconstructed catheter position could be registered in the image from the CT scanner, which would lead to increased safety and efficiency of the treatment. 

## Figures and Tables

**Table 1:** Dielectric parameters of materials and tissues used in numerical models, taken from [34].

Material (Tissue)	Frequency Band 1–4 GHz ($f_{c}=2.5\;GHz$)		Frequency Band 1–6 GHz ($f_{c}=3.5\;GHz$)		Frequency Band 1–10 GHz ($f_{c}=5.5\;GHz$)	
	Relative Permittivity $\varepsilon_{r}$ (-)	Electrical Conductivit σ (S/m)	Relative Permittivity $\varepsilon_{r}$ (-)	Electrical Conductivity σ (S/m)	Relative Permittivity $\varepsilon_{r}$ (-)	Electrical Conductivity σ (S/m)
Adrenal Gland	58.0	1.08	57.2	1.27	56.3	1.65
Blood Wessel	42.5	1.47	41.2	2.19	38.6	4.03
Cartilage	38.7	1.79	36.6	2.65	32.7	4.59
Fat	10.8	0.28	10.5	0.42	9.94	0.77
Gallbladder	57.6	2.10	56.6	2.96	54.1	5.31
Kidney	52.6	2.47	50.6	3.35	47.2	5.53
Liver	43.0	1.72	41.4	2.47	38.6	4.33
Matching Liquid	43.0	0.10	41.4	0.10	38.6	0.10
Muscle	52.7	1.77	51.4	2.56	48.9	4.61
RFA Catheter	PEC *	PEC *	PEC *	PEC *	PEC *	PEC *
Skin	38.0	1.49	37.0	2.02	35.4	3.46
Thorax Cancellous	18.5	0.82	17.4	1.20	15.6	2.02
Thorax Cortical	11.4	0.40	10.8	0.62	9.81	1.08

* Perfect Electrical Conductor. Set up within the numerical simulations in Sim4Life.

**Table 2:** Maximum and minimum dimensions of the used rectangular grid.

Dimension	Rectangular Grid		
	X-Axis	Y-Axis	Z-Axis
Number of cells (-)	533	556	153
Minimum cell size (mm)	0.096	0.154	0.214
Maximum cell size (mm)	0.981	0.998	0.984
Total number of voxels (-)	45.341 M		

**Table 3:** Detection accuracy-UWB pulse frequency band optimization test.

Frequency band (GHz)	1–4	1–6	1–10
**Detection accuracy** $d_{max}$ **(mm)**	17.38	2.16	3.74

**Table 4:** Detection accuracy-Optimized heterogeneous model.

Position	1	2	3	Mean ± STD
**Detection accuracy** $d_{max}$ **(mm)**	2.16	2.99	3.00	2.72 ± 0.48

**Table 5:** Detection accuracy of the catheter position detection during insertion.

Insertion Step (-)	1	2	3	4	5	6	7	8	Mean ± STD
$d_{centr}$ (mm)	4.24	1.41	0.95	3.53	3.20	4.31	4.90	4.11	3.33 ± 1.43

**Table 6:** Comparison of the average relative permittivity determined by the radar with the relative permittivity of significant tissues in the imaged area.

Tissue	Relative Permittivity $\varepsilon_{r}$ (-)		
	Frequency Band 1–4 GHz ($f_{c}=2.5\;GHz$)	Frequency Band 1–6 GHz ($f_{c}=3.5\;GHz$)	Frequency Band 1–10 GHz ($f_{c}=5.5\;GHz$)
**Liver**	43.0	41.4	38.6
**Muscle**	52.7	51.4	48.9
**$\bar{\varepsilon_{r}}$ (TOF method)**	49.6	45.5	40.6

**Table 7:** Detection accuracy-Comparison of the accuracy achieved within the numerical model and measurement.

Position (-)	Detection Accuracy $d_{max}$ (mm)	
	Homogeneous Model (Numerical Study)	Experimental Model (Measurement)
**1**	3.78	6.20
**2**	3.77	6.75
**3**	4.10	5.43
**Mean ± STD**	3.88 ± 0.19	6.13 ± 0.66

## Data Availability

The data presented in this study are available on request from the corresponding author.

## References

[1] Siegel R.L., Miller K.D., Jemal A. (2020). Cancer statistics, 2020. CA A Cancer J. Clin..

[2] Jemal A., Ward E., Thun M., Miranda J.J. (2010). Declining Death Rates Reflect Progress against Cancer. PLoS ONE.

[3] Stauffer P.R., Goldberg S.N. (2009). Introduction: Thermal ablation therapy. Int. J. Hyperth..

[4] Kok H.P., Cressman E.N.K., Ceelen W., Brace C.L., Ivkov R., Grüll H., ter Haar G., Wust P., Crezee J. (2020). Heating technology for malignant tumors: A review. Int. J. Hyperth..

[5] Tammam E., Said A.M., Ibrahim A.A., Galal A.I.A. (2020). About the Interstitial Microwave Cancer Ablation: Principles, Advantages and Challenges. IEEE Access.

[6] Cho Y.K., Rhim H., Noh S. (2011). Radiofrequency Ablation versus Surgical Resection as Primary Treatment of Hepatocellular Carcinoma Meeting the Milan Criteria: A Systematic Review. J. Gastroenterol. Hepatol..

[7] Simon C.J., Dupuy D.E., Mayo-Smith W.W. (2005). Microwave Ablation: Principles and Applications. RadioGraphics.

[8] Brace C.L. (2009). Microwave Ablation Technology: What Every User Should Know. Curr. Probl. Diagn. Radiol..

[9] Sjølie E., Langø T., Ystgaard B., Tangen G.A., Nagelhus Hernes T.A., Mørvik R. (2003). 3D ultrasound-based navigation for radiofrequency thermal ablation in the treatment of liver malignancies. Surg. Endosc..

[10] Takayasu K., Muramatsu Y., Asai S., Muramatsu Y., Kobayashi T. (1999). CT fluoroscopy-assisted needle puncture and ethanol injection for hepatocellular carcinoma: A preliminary study. Am. J. Roentgenol..

[11] Abi-Jaoudeh N., Kruecker J., Kadoury S., Kobeiter H., Venkatesan A.M., Levy E., Wood B.J. (2012). Multimodality Image Fusion–Guided Procedures: Technique, Accuracy, and Applications. Cardiovasc. Interv. Radiol..

[12] Wood B.J., Kruecker J., Abi-Jaoudeh N., Locklin J.K., Levy E., Xu S., Solbiati L., Kapoor A., Amalou H., Venkatesan A.M. (2010). Navigation Systems for Ablation. J. Vasc. Interv. Radiol..

[13] Kim Y.J., Lee M.W., Park H.S. (2013). Small hepatocellular carcinomas: Ultrasonography guided percutaneous radiofrequency ablation. Abdom. Imaging.

[14] Lee M.W. (2014). Fusion imaging of real-time ultrasonography with CT or MRI for hepatic intervention. Ultrasonography.

[15] Maybody M., Stevenson C., Solomon S.B. (2013). Overview of Navigation Systems in Image-Guided Interventions. Tech. Vasc. Interv. Radiol..

[16] Sánchez Y., Anvari A., Samir A.E., Arellano R.S., Prabhakar A.M., Uppot R.N. (2017). Navigational Guidance and Ablation Planning Tools for Interventional Radiology. Curr. Probl. Diagn. Radiol..

[17] Nawfel R.D., Judy P.F., Silverman S.G., Hooton S., Tuncali K., Adams D.F. (2000). Patient and Personnel Exposure during CT Fluoroscopy-guided Interventional Procedures. Radiology.

[18] Kloeckner R., dos Santos D.P., Schneider J., Kara L., Dueber C., Pitton M.B. (2013). Radiation exposure in CT-guided interventions. Eur. J. Radiol..

[19] Fiser O., Helbig M., Sachs J., Ley S., Merunka I., Vrba J. (2018). Microwave Non-Invasive Temperature Monitoring Using UWB Radar for Cancer Treatment by Hyperthermia. Prog. Electromagn. Res..

[20] Skolnik M.I. (1990). Radar Handbook.

[21] Scapaticci R., Lopresto V., Pinto R., Cavagnaro M., Crocco L. (2018). Monitoring Thermal Ablation via Microwave Tomography: An Ex Vivo Experimental Assessment. Diagnostics.

[22] Bucci O.M., Cavagnaro M., Crocco L., Lopresto V., Scapaticci R. Microwave ablation monitoring via microwave tomography: A numerical feasibility assessment. Proceedings of the 10th European Conference on Antennas and Propagation (EuCAP).

[23] Wang M., Crocco L., Costanzo S., Scapaticci R., Cavagnaro M. (2022). A Compact Slot-Loaded Antipodal Vivaldi Antenna for a Microwave Imaging System to Monitor Liver Microwave Thermal Ablation. IEEE Open J. Antennas Propag..

[24] Helbig M., Dahlke K., Hilger I., Kmec M., Sachs J. (2012). UWB microwave imaging of heterogeneous breast phantoms. Biomed. Eng. Biomed. Tech..

[25] Kwon S., Lee S. (2016). Recent Advances in Microwave Imaging for Breast Cancer Detection. Int. J. Biomed. Imaging.

[26] Wörtge D., Moll J., Krozer V., Bazrafshan B., Hübner B., Park C., Vogl T. (2018). Comparison of X-ray-Mammography and Planar UWB Microwave Imaging of the Breast: First Results from a Patient Study. Diagnostics.

[27] Ley S., Sachs J., Faenger B., Hilger I., Helbig M. (2021). MNP-Enhanced Microwave Medical Imaging by Means of Pseudo-Noise Sensing. Sensors.

[28] Shin H.J., Narayanan R.M., Asmuth M.A., Rangaswamy M. (2016). Ultrawideband Noise Radar Tomography: Principles, Simulation, and Experimental Validation. Int. J. Microw. Sci. Technol..

[29] Scapaticci R., Di Donato L., Catapano I., Crocco L. (2012). A Feasibility Study on Microwave Imaging for Brain Stroke Monitoring. Prog. Electromagn. Res. B.

[30] Merunka I., Massa A., Vrba D., Fiser O., Salucci M., Vrba J. (2019). Microwave Tomography System for Methodical Testing of Human Brain Stroke Detection Approaches. Int. J. Antennas Propag..

[31] Conceição R.C., Mohr J.J., O’Halloran M. (2016). An Introduction to Microwave Imaging for Breast Cancer Detection.

[32] Fiser O., Hruby V., Merunka I., Vrba J. Numerical Study of Stroke Detection Using UWB Radar. Proceedings of the 2018 Progress in Electromagnetics Research Symposium (PIERS-Toyama).

[33] ROGERS CORPORATION (2018). RO4000® Series High Frequency Circuit Materials. https://rogerscorp.com/-/media/project/rogerscorp/documents/advanced-electronics-solutions/english/data-sheets/ro4000-laminates-ro4003c-and-ro4350b---data-sheet.pdf.

[34] Hasgall P.A., Di Gennaro F., Baumgartner C., Neufield E., Lloyd B., Gosselin M.C., Payne D., Klingenböck A., Kuster N. (2022). IT’IS Database for Thermal and Electromagnetic Parameters of Biological Tissues.

[35] IT’IS Foundation (2022). IT’IS Foundation.

[36] Karabulut K., Aucejo F., Akyildiz H.Y., Siperstein A., Berber E. (2012). Resection and radiofrequency ablation in the treatment of hepatocellular carcinoma: A single-center experience. Surg. Endosc..

[37] Berber E., Herceg N.L., Casto K.J., Siperstein A. (2004). Laparoscopic radiofrequency ablation of hepatic tumors: Prospective clinical evaluation of ablation size comparing two treatment algorithms. Surg. Endosc..

[38] AngioDynamics (2013). StarBurst MRI: Tech Sheet. https://www.angiodynamics.com/wp-content/uploads/2020/10/StarBurst_MRI_Tech_Sheet-071945.pdf.

[39] Fiser O., Hruby V., Vrba J., Drizdal T., Tesarik J., Vrba J., Vrba D. (2022). UWB Bowtie Antenna for Medical Microwave Imaging Applications. IEEE Trans. Antennas Propag..

[40] Zhang R., Wu S., Wu W., Gao H., Zhou Z. (2019). Computer-assisted needle trajectory planning and mathematical modeling for liver tumor thermal ablation: A review. Math. Biosci. Eng..

